# Sodium Dodecyl Sulfate Modified Carbon Nano Tube Paste Electrode for Sensitive Cyclic Voltammetry Determination of Isatin

**DOI:** 10.34172/apb.2021.012

**Published:** 2020-11-07

**Authors:** Amrutha Balliamada Monnappa, Jamballi Gangadharappa Manjunatha, Aarti Sripathi Bhatt, Kodi Malini

**Affiliations:** ^1^Department of Chemistry, FMKMC College, Madikeri, Constituent College of Mangalore University, Karnataka, India.; ^2^Department of Chemistry, N.M.A.M. Institute of Technology, (Visvesvaraya Technological University, Belgavi) Nitte, 574110, Udupi District, Karnataka, India.; ^3^Department of Chemistry, St Philomina College, Puttur, Dakshina Kannada, Karnataka, India.

**Keywords:** Cyclic voltammetry, Carbon nanotube paste electrode, SDS, Isatin, Resorcinol

## Abstract

***Purpose:*** Isatin (IS) is a synthetically significant heterocyclic moiety with an influential pharmacodynamic indole nucleus and hence the electrocatalytic property of has been investigated.

***Methods:*** The electrochemical analysis was demonstrated by cyclic voltammetry (CV) in the potential window of 0.2 V to 1.4 V using sodium dodecyl sulfate (SDS) modified carbon nano tube paste electrode (SDSMCNTPE) over a pH range of 6 to 8.5 in 0.2 M phosphate buffer solution (PBS). Surface morphology was studied by using Field emission-scanning electron microscopy (FESEM).

***Results:*** The CV study discloses that under ideal condition oxidation of IS arises at a potential of 0.970 V accompanied with an exceptional stability, selectivity and sensitivity for the resultant SDSMCNTPE contrasting to bare carbon nano tube paste electrode (BCNTPE). Individual parameters like electrode surface area, effect of surfactant, detection limit, simultaneous detection of IS and resorcinol (RC) were studied at a scan rate of 0.1 V/s. Scan rate study uncovers the process is diffusion controlled. The oxidation peak current amplified linearly with the surge in concentration of IS under ideal condition. Detection limit (LOD) and limit of quantification (LOQ) in the solution of optimum pH (7.5) at a scan rate of 0.100V/s is 2.4×10^-7^ M and 8.2 × 10^-7^ M respectively.

***Conclusion:*** The proposed electrode portrays excellent repeatability, reproducibility and reliability to resistant electrode fouling.

## Introduction

Heterocyclic compounds are an important class of organic compounds which possess a cyclic structure with at least atoms of two different elements as members of its rings. Isatin (IS) i.e. indole 2,3 dione [C_8_H_5_NO_2_] is one such synthetically versatile heterocyclic moiety which is used in the preparation of different IS derivatives, which possess rich biological and pharmacological properties. IS and its derivatives show various biological activities like anti-cancer, anti-malarial, anti-tubercular, analgesic, anti-bacterial, antifungal, anti-diabetic, anti-convulsant, anti-HIV, anti-inflammatory, and anti-anxiety.^1-3^ IS and its derivatives are widely used in industries as corrosion inhibitors, as fluorescent sensors and in the dye industry.

**Figure 1:**
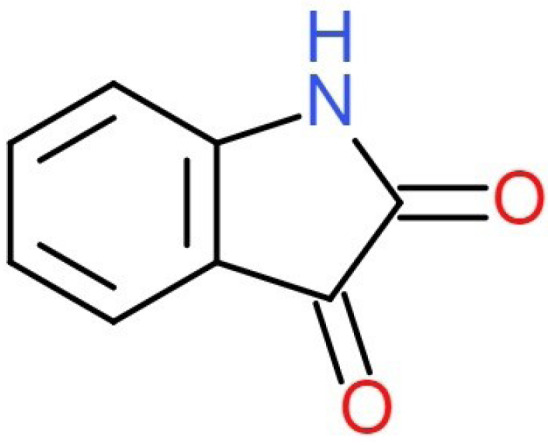
Structure of IS.

IS acts as an effective endogenous neurochemical regulator in the brain of mammals, since it is a metabolic derivative of adrenaline and mammalian tissue.^4,5^ It also acts as discerning inhibitor of monoamine oxidase B which is used as antidepressants for patients with Parkinson’s ailment. With the severity of disease, the concentration of IS is found to have a noticeable increase in the urine sample of the patients. The natural source of IS is the parotid gland secretion of the Bufo frog and few plants species like genus *Isatis*. In rat model IS levels in brain, heart and blood plasma is found to increase with stress. Different range of behavioural changes will be observed depending on the dose of IS administered by in vivo method, which comprises anxiety if low dose (10-20 mg/kg) is administered and sedation if high dose (80-200 mg/kg) is administered.^6,7^ These diverse effects suggest that different biochemical mechanisms are involved and detection of IS is of potential importance. Since IS is an electrochemically active moiety which can be detected voltammetrically because of its high sensitivity voltammetric technique provides admirable insights about the oxidation mechanism of this moiety.^8^ Structure of IS is depicted in Figure 1.

Carbon nanotubes (CNTs) also called buckytubes is composed of a concentric arrangement of several cylinders which has occurred as one of the most vigorously studied nanostructured materials. Owing to their exceptional amalgamation of mechanical, chemical, optical and electrical properties they serve as a hopeful tool in biosensing. Moreover, CNTs also serve as platforms to conjugate other compounds at their surface by immobilization of functional units, since they have a bulky specific surface area and consistent active sites. CNTs are extensively used in cyclic voltammetry (CV) studies due to their aptitude to provide low detection limit, antifouling property, high sensitivity and diminished overpotential. The stability and responsiveness of the CNT’s can be enhanced with suitable surfactant since they have superior electroanalytical properties.^9-12^

Sodium dodecyl sulfate (SDS), is an anionic surfactant with a head which has a strong affinity to water on one side and a long tail which repels water on the opposite side. They get adsorbed on the electrode surface and aggregates the electron allocation along with moderately improving the peak current. SDS will interact with the electroactive species molecules through electrostatic force of attraction and encourages the electron transmission between the electrodes and the electroactive species in the solution, thereby improving the selectivity and sensitivity of the analysis.^13^ A modest, swift and sensitive CV system for the determination of IS at an SDS modified carbon nano tube paste electrode (SDSMCNTPE) is proposed in this study. The experimental outcome demonstrates that the surfactant SDS has a discrete augmentation impact on the electrochemical reciprocation of IS. Examination of the electrochemical parameters in IS oxidation were conducted.^14,15^

## Materials and Methods

### Apparatus

CV was conducted by using electrochemical analyser model CHI6038E [CH- Instrument from USA] with a conventional three electrode cell and connected to a desktop for storage of data. Bare carbon nano tube paste electrode (BCNTPE), SDSMCNTPE as working electrode, calomel electrode as reference electrode and a platinum wire served as counter electrode.

### Chemicals and Reagents

IS, SDS, Triton X -100 (TX-100), Cetyl trimethylammonium bromide (CTAB), Graphite, Monosodium dihydrogen phosphate, Disodium hydrogen phosphate and Silicone oil were purchased from Nice chemicals, Cochin, India. Acetone and resorcinol (RC) from Molychem, Mumbai, India. Multi-walled Carbon Nanotube (MWCNT) with a measurement of 30-50 nm and a length of 10-30 µm was acquired from Sisco research laboratories in Maharashtra. 25 × 10^-4^ M stock solutions of SDS, TX-100, CTAB were prepared in double distilled water. IS (1×10^-3^ M) stock solution was prepared by dissolving it in Acetone and RC (1×10^-3^ M) was dissolved in double distilled water. Phosphate buffer solution (PBS) solution of strength 0.2 M used as a supporting electrolyte was prepared by inter mixing the required quantity of 0.2 M NaH_2_PO_4_ and 0.2 M Na_2_HPO_4_. Analytical grade chemicals were utilised with no additional refinement.

### Preparation of BCNTPE and SDS/MCNTPE

Carbon nanotube paste electrode (CNTPE) was prepared by optimising the ratio of CNT powder to the binder. CNT powder and silicone oil were mixed in the proportion of 60:40 (w/w) thoroughly in the agate mortar using a pestle to get a consistent mixture. The resulting homogeneous paste was tightly packed into the cave of 3 mm diameter Teflon tube and rubbed on the smooth weighing paper to get a smooth surface of the BCNTPE. A copper wire implanted into the Teflon tube develops contact with the external circuit. 10 µl of surfactant SDS was immobilised onto the surface of BCNTPE by drop coating method and left for 5 minutes so that maximum adsorption of the surfactant on the electrode surface takes place and later the unreacted residue of surfactants was rinsed with double distilled water.

## Results and Discussion

### Characterisation of BCNTPE and SDSMCNTPE 

**Figure 2:**
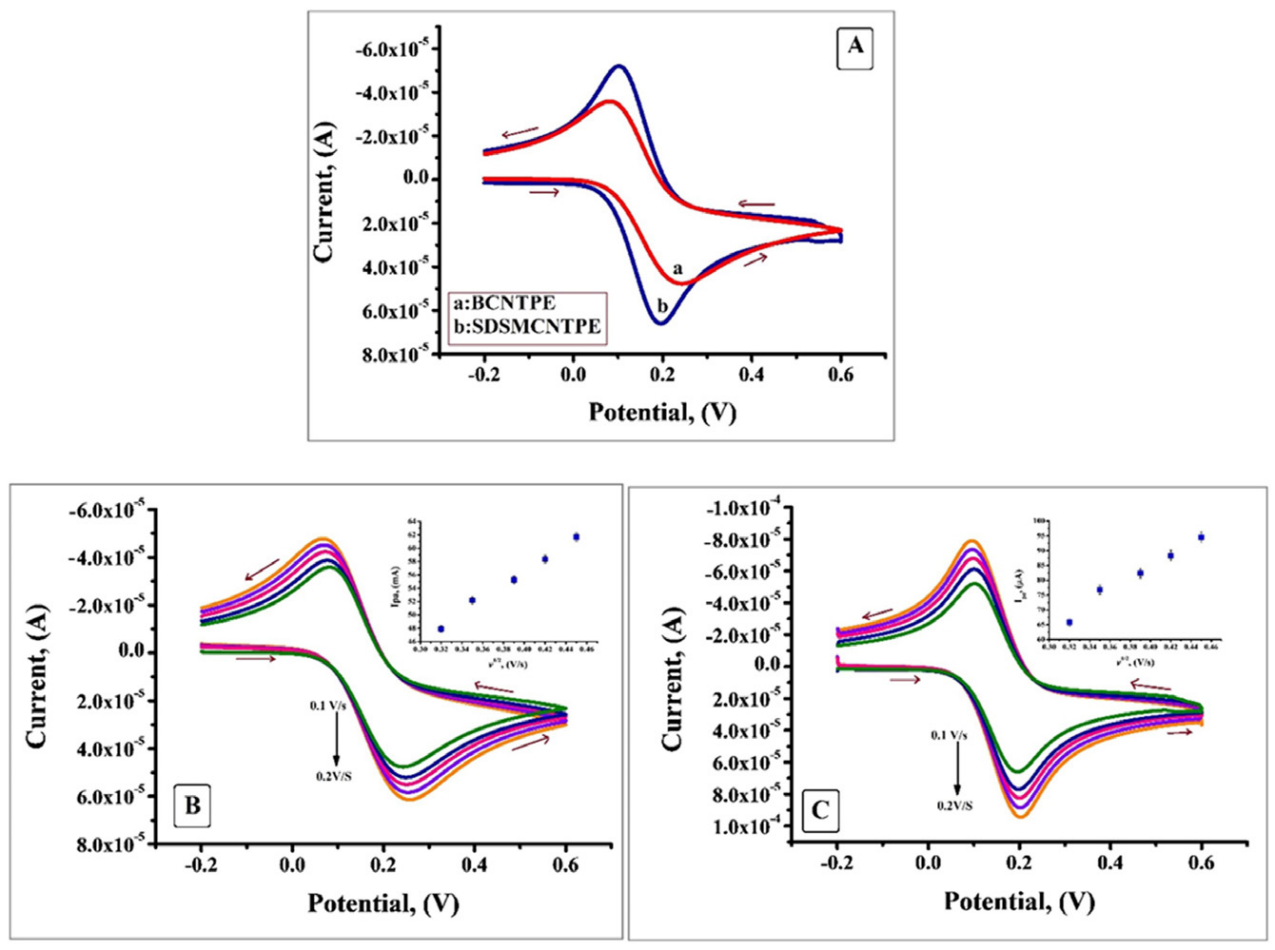
A) Cyclic voltammograms of 0.2 mM K4[Fe (CN)6] at BCNTPE and SDSMCNTPE in 0.1M KCl at the scan rate of 0.1V/s. B) Cyclic voltammograms obtained for BCNTPE in the same electrolytic solution at different scan rates (0.1 V/S to 0.2 V/s). C) Cyclic voltammograms obtained for SDSMCNTPE in the same electrolytic solution at different scan rates (0.1 V/S to 0.2 V/s).

The electrochemical properties of SDSMCNTPE was investigated by using K_4_[Fe (CN)_6_] as the electrochemical redox probe. Figure 2A portrays the cyclic voltammogram obtained for the oxidation of 0.2 mM K_4_[Fe (CN)_6_] at BCNTPE and SDS/MCNTPE in the presence of 0.1M KCl as supporting electrolyte at a scan rate of 0.1 V/s. From the CV obtained it is clear that low redox peak current response was observed at BCNTPE but under similar condition SDSMCNTPE revealed static augmentation in redox peak currents which is attributed to the increase in electrochemical active sites and the electrocatalytic property of SDS. The difference between E_pa_ and E_pc_ (ΔE_p_) at SDSMCNTPE is 0.094V which is smaller than that at BCNTPE where ΔEp is 0.161V, suggests the fact that SDS facilitates fast electron transfer due to the chemical interaction between SDS with K_4_[Fe (CN)_6_]. Since ΔE_p_ is a characteristic of rate of electron transfer the values found implies that lesser the ΔE_p_ value higher will be the electron transfer rate.^16,17^

The cyclic voltammogram was studied thoroughly at a series of scan rates from 0.1 V/s to 0.2 V/s. Figure 2B displays voltammogram of K_4_[Fe (CN)_6_] at BCNTPE where the redox peak current surges with the rise in scan rate. The plot of I_pa_ v/s square root of scan rate (Figure in the inset of Figure 2B) shows good linearity with correlation coefficient value R^2^= 0.9959. Similarly Figure 2C portrays the voltammogram of K_4_[Fe (CN)_6_] at SDSMCNTPE under identical condition where the redox peak current shows enhancement with the rise of scan rate. The plot of I_pa_ v/s square root of scan rate (Figure in the inset of Figure 2C) shows linearity with R^2^= 0.9898. Scan rate studies reveal that the process is diffusion controlled. By using K_4_[Fe (CN)_6_] as a probe at various scan rates the electroactive surface area of BCNTPE and SDSMCNTPE were calculated by using Randles-Sevcik formula:

I_pa_ =2.69 × 10^5^ n ^3/2^ A Co D ^1/2^*v*^½^(Eq.1)

I_pa_ is the oxidation peak current (µA), n =1 represents number of electron exchanged, C_o_ (mol cm^-3^) represents concentration of electroactive species, D =7.6 ×10^-6^ cm^2^ s^-1^ is diffusion coefficient, *v*^½^ is square root of scan rate, the active surface area ‘A’ was calculated from the slope of the plot of I_pa_ v/s *v*^½^. For SDSMCNTPE the electroactive surface area is maximum (0.0 45 cm^2^) as compared with BCNTPE (0.022 cm^2^).

**Figure 3:**
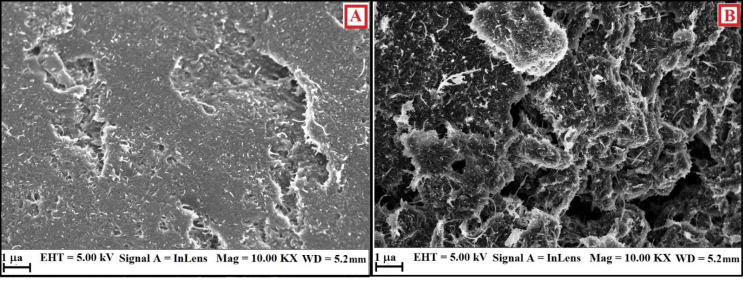
A) FESEM depiction of BCNTPE. B) FESEM depiction of SDSMCNTPE.

More evidence for the modification of BCNTPE was attained from the surface characterisation studies using Field emission scanning electron microscopy (FESEM). Figure 3A reveals that the surface of BCNTPE is of irregular shape (A) with less available surface area. After the surface modification the electrode surface portrays agglomerated morphology, stipulating the alteration of the electrode surface, with spongy surface (Figuer 3B). Hence, it is confirmed that the surface morphology of SDSMCNTPE has been significantly altered and leads to contented oxidation of targeted molecules.^18,19^

**Figure 4:**
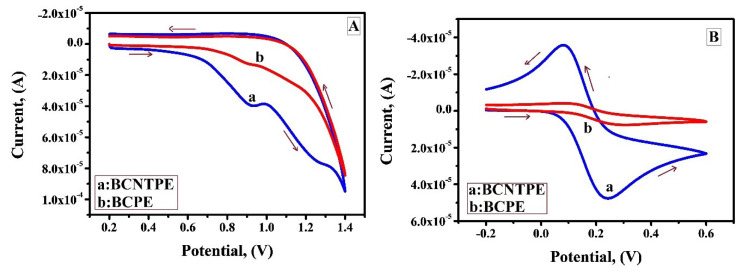
A) Cyclic Voltammogram proﬁle of 1×10^-3^ M IS at BCNTPE (curve a) and BCPE (curve b) in 0.2 M PBS (pH 7.5) at a scan rate of 0.1 V/s. B) Cyclic voltammograms of 0.2 mM K_4_[Fe (CN)_6_] at BCNTPE (curve a) and BCPE (curve b) in 0.1M KCl at the scan rate of 0.1V/s

Few studies reveal that IS and its derivatives are electrochemically oxidised at carbon paste electrodes. Figure 4A portrays the CV curves for the electrochemical behaviour of bare carbon paste electrode (BCPE) and BCNTPE in oxidation of IS by using 0.2 M PBS as supporting electrolyte. Remarkably, it tends to be seen effectively that BCNTPE (curve a) shows enhanced current sensitivity indicating the improved electron transfer in comparison with BCPE (curve b). The enhanced current sensitivity in BCNTPE can be attributed to soaring conductivity, consistent active sites which boosts their electrocatalytic activity in comparison with BCPE. It is because of this perception BCNTPE was selected for subsequent work. The active surface area of BCNTPE and BCPE was examined by the application of CV method in 0.2 mM K_4_[Fe (CN)_6_] with 0.1 M KCl at a scan rate of 0.1 V/sec. As expected, Figure 4B shows that compared to peak current at BCPE (curve b), peak current at BCNTPE (curve a) shows enhancement and this clarifies the fact that apparent electrochemical area of BCPE exposed to the solution is smaller than the electrochemical area of BCNTPE.^20,21^

### Effect of pH 

**Figure 5:**
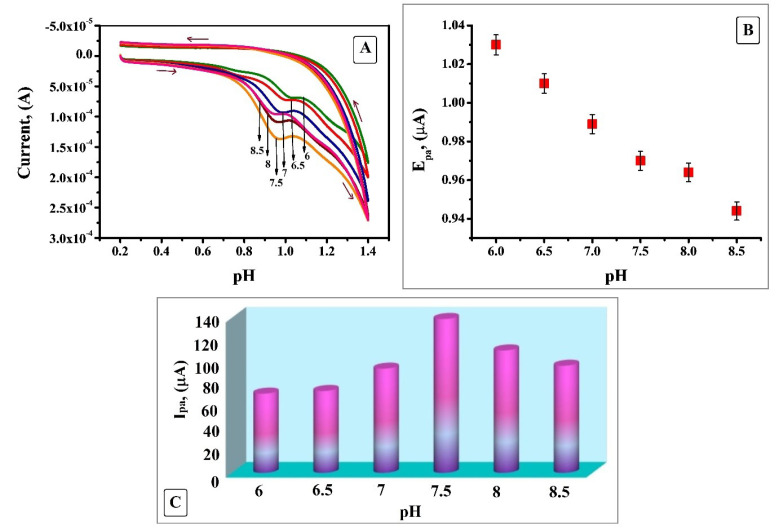
**A)** Cyclic Voltammogram obtained for influence of supporting electrolyte of 0.2 M PBS on 1 × 10^-3^ M IS at SDSMCNTPE with a scan rate of 0.1 V/s. B) Plot of E_pa_ Vs pH at SDSMCNTPE. C) Plot of I_pa_ Vs pH at SDSMCNTPE.

The pH of a solution is a significant factor which will have a robust influence on the electrochemical oxidation behaviour of an analyte at the electrode surface. Sharper response escorted with higher sensitivity can be attained by optimising the pH of the solution. The effect of pH in the range of 6 to 8.5 at the SDSMCNTPE electrode by using 0.2 M PBS as supporting electrolyte at a scan rate of 0.1 V/s (Figure 5A) was considered. Hence from the figure it is clear that with the rise of pH the anodic peak potential is displaced to further negative values due to the hindrance of oxidation at lesser proton concentration (Figure 5B).^22^ The relationship is linear over the entire pH range with a linear regression equation E_pa_ (V) = 1.2234 - 0.033 pH and (R^2^ = 0.9857) where R^2^ is correlation coefficient. The plot of the variation of anodic peak current I_pa_ vs pH (Figure 5C) shows that the peak current surges with the rise in pH, reaches an optimum value at 7.5 and a steady decrease was noticed after that. Due to faster electron transfer and active interaction amid IS and SDSMCNTPE highest current response with improved sensitivity and reliable profile of voltammogram was obtained at pH 7.5. Hence, this electrolyte was chosen as a sustaining electrolyte for subsequent studies.^23^

### Optimization of the modifier on the electrode 

**Figure 6:**
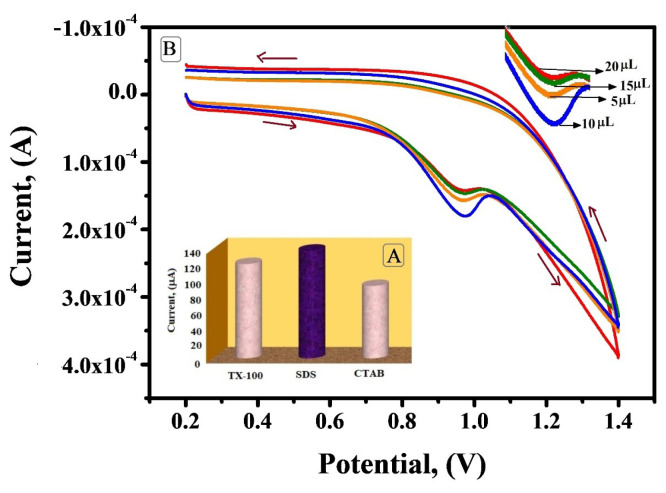
A) Plot of different surfactants (TX-100, SDS, CTAB) v/s I_pa_ (µA) (Figure in the inset). B) Cyclic voltammograms (*v* =0.1V/s) of IS (1×10^-3^ M) at SDSMCNTPE in 0.2 M PBS, pH 7.5, for different amounts of SDS (5-20 µL)

The voltammetric response of IS was reliant on the type and amount of surfactant immobilised on the surface of BCNTPE. The peak current intensity for IS oxidation was analysed by using different surfactants like SDS, cetyltrimethylammonium bromide (CTAB) and Triton X-100 (TX-100). Among these surfactants SDS exhibited enhanced peak current in contrast to CTAB and TX-100 (Figure 6A in the inset). These results justify the fact that anionic surfactant like SDS can promote electrochemical oxidation because of its head group and alkyl chain length. Surfactants have appealing property of accumulating substrates with unlike charges and repelling the species with like charges. In the optimum pH range IS exists in the cationic state so the anionic surfactant will enhance the accumulation of molecule which is in cationic form. But CTAB resist IS and Triton X-100 may hamper the formation of adsorption layer. Hence SDS was preferred for subsequent investigation.^24,25^

The relationship between the volume of SDS immobilised on the surface of BCNTPE and anodic peak current was determined. The oxidation peak current of IS increased with the increase in volume of SDS dropped on BCNTPE, where at 10 µL it showed enhanced peak current and after that with the further increase in volume the peak current decreased significantly (Figure 6B). When the volume of SDS surfactant immobilised on the BCNTPE surface was too small, the amount of adsorbed IS was also small and subsequently the peak current was small. When the immobilised volume of surfactant was too large, the peak current contrarywise showed gradual decline, probably attributed to the fact of critical aggregation concentration. Hence 10 µL of SDS was considered as the optimum value to modify BCNTPE to SDSMCNTPE.^26,27^

### Electrochemical behaviour of IS at SDSMCNTPE

**Figure 7:**
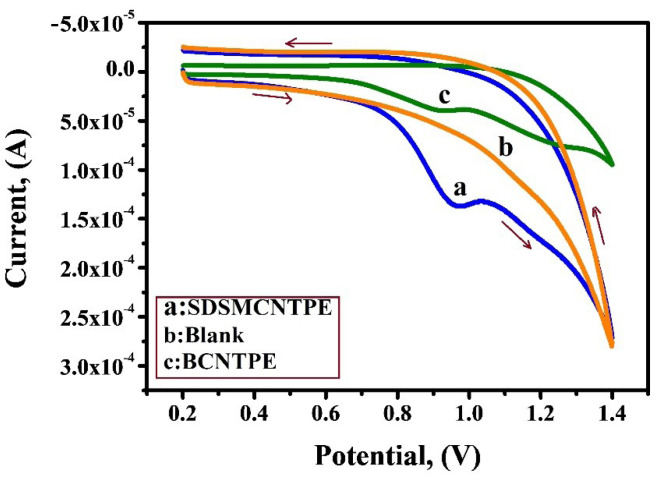
Comparative study of 1×10-3 M IS at SDSMCNTPE (curve a), In the absence of IS (curve b) and BCNTPE (curve c) in 0.2 M PBS of pH 7.5 at a scan rate of 0.1 V/s.

To highlight the appealing behaviour of SDSMCNTPE in the electrochemical oxidation of IS under diverse circumstances, the CV was equated at a scan rate of 0.1 V/s in the potential window of 0.2 V to 1.4 V. Figure 7 displays cyclic voltammograms of IS at pH 7.5 in 0.2 M phosphate buffer at SDSMCNTPE (curve a), blank solution (curve b) and at BCNTPE (curve c). At BCNTPE (curve c) 1 × 10^-3^ MIS shows poor voltammetric response and low current signal of 40.04 µA due to the low rate of electron transfer. On the contrary the voltammogram obtained under identical condition for SDSMCNTPE (curve a) gives a well resolved peak at a potential of 0.970 V with enhanced current signal of 138.8 µA due to augmentation in surface area. The presence of oxidation peak for SDSMCNTPE establishes the modification of BCNTPE by a thin film of SDS which remarkably boosts the affinity of modified electrode in the oxidation of IS. Since no reduction peak is detected while reverse scanning it is obvious that the electrode response is an irreversible process. From curve b it is obvious that the oxidation peak current and oxidation peak potential responses were not revealed in the absence of IS (curve b) but under alike condition, in the presence of IS (curve a), an improved voltammetry response is observed.^28-30^

### Calibration plot and influence of concentration of IS on the peak current

**Figure 8:**
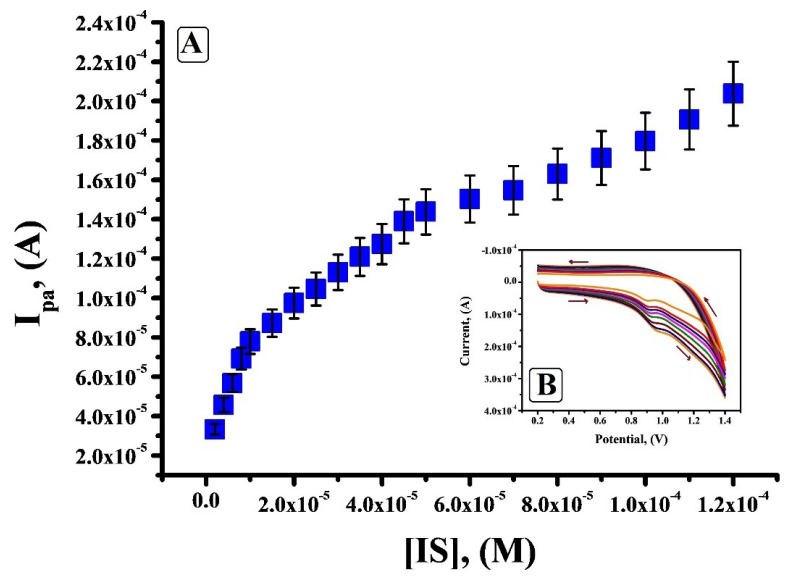
A) Calibration plot (v =0.1V/s) for the determination of IS at SDSMCNTPE in 0.2 M PBS (pH 7.5), B) Cyclic voltammogram for variation of concentration of 1×10-3 M IS in 0.2 M PBS of pH 7.5 at SDSMCNTPE.

Theconcentration of IS was varied from 2 × 10^-6^ M to 1.2 × 10^-4^ M and according to the electrochemical reply obtained from Figure 8B, it is clear that under optimal condition the peak current amplified linearly with the surge in concentration of IS and a small shift in the oxidation potential towards positive side was observed. Figure 8A shows three linear ranges 2×10^-6^ M to 1×10^-5^ M, 1.5×10^-5^ Mto 5×10^-5^ M and 6×10^-5^ M to 1.2×10^-4^ M. The first linear range with a linear regression equation I_pa_ (µA) = 2.29 × 10^-5^ + 5.582 C (M) and correlation coefficient of 0.9978 was considered. The calculated values of limit of detection (LOD) and limit of quantification (LOQ) was 0.24 × 10^-6^ M and 0.82 × 10^-6^ M respectively.

($LOD=\frac{3S}{N}\;and\;LOQ=\frac{10S}{N}$ where S is the Standard deviation, and N is the slope)^31^

Comparison of detection limit for determination of IS with some modified electrodes is tabulated in Table 1.

**Table 1:** Comparison of LOD values of different electrodes

**Electrode**	**Method**	**LOD (M)**	**Ref.**
GCE	DPV	2.0 ×10^-7^	32
Boron doped diamond macroelectrode	CV	2.2 × 10^-7^	33
Boron-doped nanocrystalline diamond thin-film electrode	Amperometry	1.0 ×10^-7^	34
SDS/MCNTPE	CV	2.4 ×10^-7^	Present work

The statistics in Table 1 shows the LOD values of SDSMCNTPE is in close approximate with the LOD values of other electrodes. Moreover, the sensor used in the present work is less expensive and modest to use, than other fabricated electrodes. The LOD value obtained in present work was compared with LOD values from other methods like HPLC and UV-Visible which was found to be in close proximity.^35^

### Influence of scan rate 

**Figure 9:**
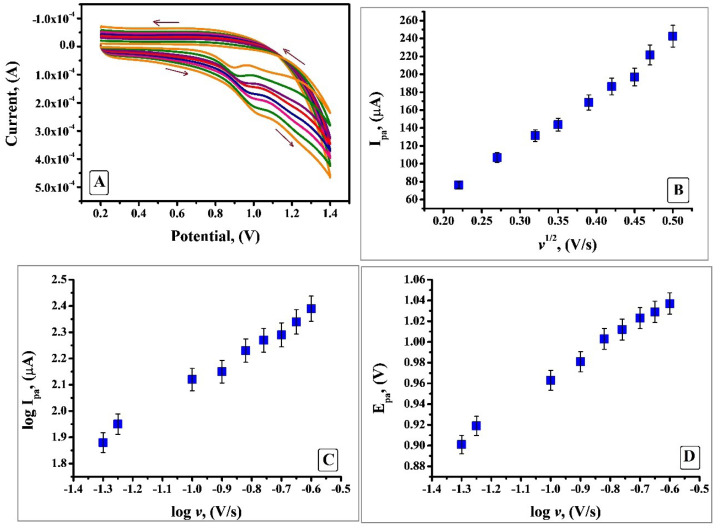
A) Cyclic voltammogram of 1×10^-3^ M IS in 0.2 M PBS of pH 7.5 at SDSMCNTPE at various scan rates (0.05 V/ s to 0.25 V/s). B) Plot of Ipa (µA) vs. square root of scan rate (*v*^1/2^). C) Plot of log I_pa_ (µA) vs log of scan rate (log *v*). D) Plot of Epa (V) vs log of scan rate (log *v*).

The evidence about the electrochemical mechanism can be attained by the relation amid anodic peak current and scan rate. Hence by utilising CV the electrochemical property of IS was studied under optimum condition by changing the scan rate from 0.05 V/s to 0.25V/s. Figure 9A shows that anodic peak current portrays enhancement with the rise in scan rate. Since no cathodic peak was observed, it confirms that the electrooxidation process of IS is irreversible. Plot of anodic peak current (I_pa_) v/s square root of scan rate (*v*^1/2^) (Figure 9B) depicts descent linear relationship with a linear regression equation I_pa_(µA) = -49.82 + 570.96 *v*^1/2^ (V/s) and R= 0.9962. Thus, it indicates that at pH 7.5 the process was characteristic diffusion controlled current system rather than adsorption controlled. Further, there was a linear relationship between log I_pa_ and log *v* (Figure 9c) which is expressed by the equation log I_pa_ (µA) = 2.2401 + 0.6430 log *v* (V/s); R=0.9975. Slope of 0.6430 is in close approximate with the ideal value of 0.5 for diffusion - controlled process.^36,37^ As it is apparent from Figure 9D the potential shifted to more positive side with an increase in scan rate which is an indication that the process involves irreversible electron transfer kinetics. For a totally irreversible process the number of electrons transferred can be calculated by using Laviron’s equation:

(Eq. 2) $$Epa=Eo+\left(\frac{2.303RT}{anF}\right)\log(\frac{RTKo}{anF})+\left(\frac{2.303RT}{anF}\right)\log v$$

**Scheme 1:**
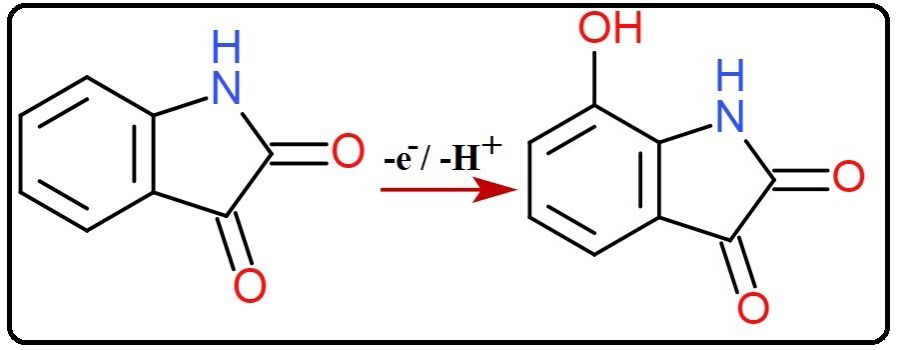
Proposed oxidation mechanism of IS.

*n* denotes the number of electrons transferred, α is the charge transfer coefficient assumed to be 0.5, k_o_ the standard rate constant of the reaction, F is Faraday constant. Rest of the symbols have their typical implication. Slope is equated to 2.303RT/αnF which is obtained from the plot of E_pa_ vs log *ν*. By substituting the particular values, the numerical value obtained for n is 1.14 and is considered as 1. Hence the oxidation reaction of IS at SDSMCNTPE proceeds through one electron transfer (Scheme 1). The estimation of electron transfer rate constant (k^o^) at the electrode electrolyte solution boundary is of paramount importance in electrochemistry and calculated using Laviron’s equation (Eq. 2) and was found to be 3.2 × 10^-4^ cm s^-1^. The value was calculated from the slope of the plot E_pa_ vs log *ν* and rest of the values have their own implication.

The average surface concentration (Γ) of IS on the surface of SDSMCNTPE was estimated to be 1.5 ×10^-8^ mol cm^-2^ based on the slope of I_pa_ vs. *ν* applying the equation:

Q = nFA Γ (Eq. 3)

Q is the amount of charge integrated from the area of cyclic voltammetric peak, n is considered to be equal to 1 and other symbols have their usual significance.^38-40^

### Electrochemical behaviour of IS and RC using SDSMCNTPE

**Figure 10:**
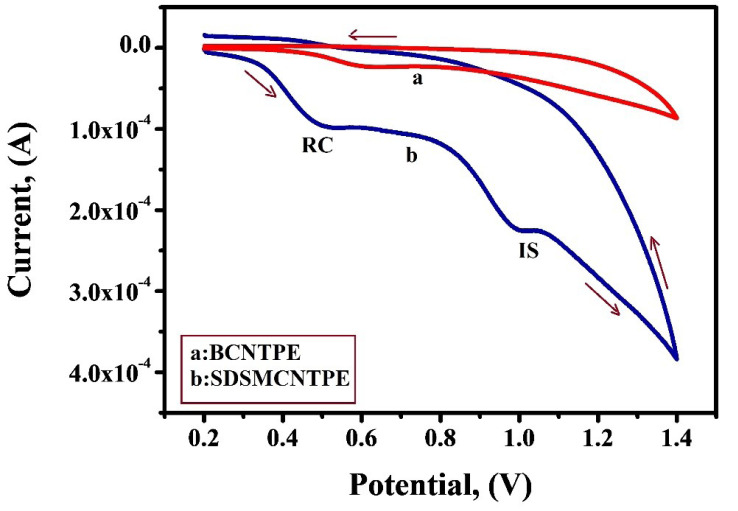
CV response of simultaneous analysis of 1×10^-3^ M IS in the presence of 1×10^-3^ M RC in 0.2 M PBS having the pH 7.5 at BCNTPE (curve a) and SDSMCNTPE (curve b) at a scan rate of 0.1 V/sec.

One of the main objectives of this study was to fabricate a sensor for detecting the electrochemical responses of RC and IS separately. Resorcinol is widely employed to treat some diseases like Psoriasis and some other skin problems. It is also used in hair dyes. IS is a very useful pharmacodynamic moiety and it is also used as a direct dye for dyeing keratin fibres especially human hair. Hence, SDSMCNTPE was used for the concurrent determination of IS (1 × 10^-3^ M) and RC (1 × 10^-3^ M) under optimized situations of pH 7.5 in 0.2 M PBS at a scan rate of 0.1V/s. The concentration of RC and IS was simultaneously changed. The voltammetric response depicted in Figure 10 shows secluded oxidation peaks with a peak parting of 0.488 V (curve b). RC showed it’s E_pa_ at 0.510V and IS at 0.998V with an enhanced current response equivalent to the oxidation of IS and RC signifying that concurrent determination of these compounds is achievable at SDSMCNTPE. On the contrary in the identical condition BCNTPE shows poor sensitivity for the concurrent determination of RC and IS (curve a).

### Examination of repeatability, reproducibility and stability

The sensing execution of the modified electrode under the ideal condition can be evaluated by parameters like repeatability, reproducibility and stability. The ability of the electrode to generate a stable, reproducible surface was observed by CV data which displays a well categorized reproducible peak. SDSMCNTPE reveals healthy reproducibility and repeatability with RSD values of 4.62 % (n=5) and 3.75% (n=4) respectively. Along with that the stability was documented after running 40 cycles the peak potential persisted unaltered, and the current signal exhibited less deterioration comparatively to the preliminary response. The percentage degradation for the fabricated electrode was calculated by using the equation:^41-43^

$$Percentage\;\deg radation=\frac{I_{pm}}{I_{pl}}\times 100$$

I_pn_ and I_p1_ are the n^th^ value and 1^st^ value of anodic peak currents. The value obtained for SDSMCNTPE was found to be 92 % which proves that fabricated electrode is decidedly stable.

## Conclusion

SDS has been immobilised on BCNTPE by immobilisation technique and the modified electrode was characterised by FESEM.The obtained results demonstrate the viability of using the fabricated electrode to the oxidation of IS. K_4_[ Fe (CN)_6_] was used as a redox probe to calculate the surface area of the SDSMCNTPE and the BCNTPE. A pair of well- defined redox peaks were obtained which increased with the rise in scan rate. Enhanced surface area was obtained for SDSMCNTPE in comparison with BCNTPE. Effect of the surfactant and the influence of various physio chemical parameters like scan rate, detection limit and pH were studied. The electrode process was found to be totally irreversible diffusion controlled with one electron transfer at an optimum pH 7.5 in 0.2 M PBS. The modified electrode portrays very good linear range with a linear regression equation I_pa_ (µA) = 2.29 × 10^-5^ + 5.582 C (M) with a LOD of 0.24 × 10^-6^ M. The proposed method presented the advantages of high sensitivity, stability, diminished fouling effect, good repeatability and simplicity.

## Ethical Issues

No ethical issues for this work.

## Conflict of Interest

No conflict of interest with any organization, reviewers and authors for this work
